# Case Report: How whole-genome sequencing-based cell-free DNA prenatal testing can help identify a marker mhromosome

**DOI:** 10.3389/fgene.2022.926290

**Published:** 2022-09-26

**Authors:** Pascale Kleinfinger, Marie Brechard, Armelle Luscan, Detlef Trost, Aicha Boughalem, Mylene Valduga, Stéphane Serero DR, Jean-Marc Costa, Laurence Lohmann

**Affiliations:** ^1^ Laboratory CERBA, Saint-Ouen-l'Aumône, France; ^2^ Cytogenetic Laboratory, Hopital Saint Joseph, Marseille, France

**Keywords:** case report, supernumerary marker chromosome, noninvasive prenatal testing, fish, array

## Abstract

A supernumerary marker chromosome (SMC) is a structurally abnormal chromosome that cannot be characterized by conventional banding cytogenetics. Marker chromosomes are present in 0.075% of prenatal cases. They are associated with variable phenotypes, ranging from normal to severely abnormal, and the prognosis is largely dependent on the results of further cytogenomic analysis. Here, we report the identification and characterization of a marker chromosome following prenatal screening in a 39-year-old pregnant patient. The patient had a normal first trimester ultrasound but was high-risk for fetal chromosome anomalies based on the results of maternal serum parameters. Chorionic villus sampling was performed, and analysis of chorionic villi revealed the presence of two identical marker chromosomes. In the interest of a rapid identification of the markers, we performed noninvasive prenatal testing (NIPT) together with chorionic villus sampling. A pericentromeric 29 Mb duplication of chromosome 20: dup (20) (p13q11.21) was identified and thereafter confirmed by targeted metaphasic FISH. Whole-genome sequencing-based NIPT was instrumental in rapid characterization of the SMCs and allowed us to obviate the need for multiple expensive and time-consuming FISH analyses.

## Introduction

A supernumerary marker chromosome (SMC) is a supplementary chromosome that cannot be characterized using conventional banding cytogenetic analysis (ISCN 2020). SMCs are usually equal in size or smaller than a chromosome 20 of the same metaphase spread (Liehr and Weise, 2007). Marker chromosomes have been shown to be present in 0.075% of unselected prenatal cases but only in 0.044% of consecutively studied postnatal cases (Liehr and Weise, 2007). The clinical phenotypes associated with marker chromosomes can be highly variable, ranging from normal to severely abnormal (Paoloni-Giacobino et al., 1998; Jang et al., 2016). The prognosis in pregnancies with marker chromosomes depends on whether euchromatin is present, if the marker chromosome is inherited or *de novo*, if it is homogeneous or mosaic, whether it is confined to the placenta, and on the presence or absence of uniparental disomy (UPD) if the marker is derived from a chromosome subjected to imprinting (Starke et al., 2003). Thus, to determine the prognosis, it is essential to characterize the SMC. There are two primary molecular cytogenetic methods used for identification and characterization of SMCs: Centromeric fluorescence *in situ* hybridization (FISH) and chromosomal microarray. Centromeric FISH allows characterization of markers originating from acrocentric chromosomes and is readily available, fast, and affordable. For markers originating from non-acrocentric chromosomes, it is an expensive and time-consuming method. Array allows only euchromatin detection, and low-level mosaicism can cause false-negative results. Thus, a normal array result is not always reassuring because of the risk for mosaicism and the implications of an undetected imprinted chromosome.

Cell-free DNA (cfDNA)-based noninvasive prenatal testing (NIPT) can screen for a range of fetal chromosome anomalies, with some approaches reporting aneuploidies on all chromosomes and large autosomal deletions/duplications (Fiorentino et al., 2017; Pescia et al., 2017; Pertile et al., 2021; Soster et al., 2021). As cfDNA originates from the cytotrophoblast, it is interrogating the genetic status of the placenta as a proxy for the fetus (Taglauer et al., 2014). The high sensitivity of NIPT implies that it can detect mosaic chromosome anomalies. In contrast to chorionic villus sampling (CVS), an invasive diagnostic technique that samples a small region of the placenta, NIPT noninvasively assesses the genetic status of the cytotrophoblast as a whole.

We report a case of a 39-year-old pregnant patient at high-risk for fetal chromosomal anomalies based on the results of maternal serum parameters. CVS and karyotyping of chorionic villi revealed two supernumerary marker chromosomes. NIPT allowed us to characterize the nature of the markers and effectively guide the choice of further genomic analyses of the chorionic villi. NIPT is a screening test that is usually carried out prior to invasive diagnostic testing. Here, on the contrary, NIPT was used as a follow-up tool to identify marker chromosomes primarily detected through invasive diagnostic testing.

## Case description

**FIGURE 1:**
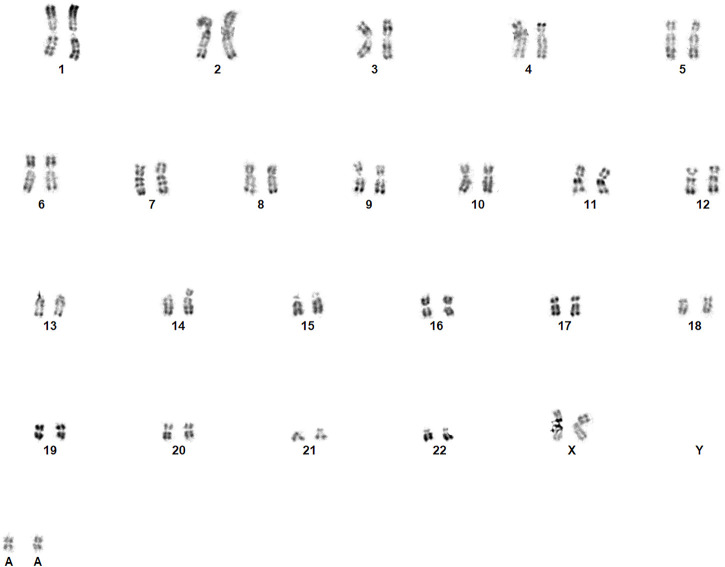
Identification of the marker chromosomes with direct examination of the cytotrophoblast following CVS [revealed two supplementary and identical SMCs (48,XX,+marx2)].

The patient was a 39-year-old pregnant woman with no relevant family history. Her obstetrical history included one voluntary termination pregnancy and two miscarriages. No medical analyses were performed to explain the miscarriages. The first trimester ultrasound at 12.6 weeks’ amenorrhea was normal (Crown Rump Length of 67.7 mm; Nuchal translucency of 2.2 mm) but maternal serum screening results from blood drawn on the same day reported the patient as being at a risk of 1:10 for trisomy 21 (β-hCG of 3.28 MoM; PAPP-A of 0.44 MoM). The patient elected to have diagnostic testing and CVS was performed at 13.1 weeks’ amenorrhea. Direct analysis of short-term cultured chorionic villi with conventional RHG banding revealed two homogeneous, supernumerary and identical SMCs: 48,XX,+marx2 (Figure 1). A genome-wide array Cytoscan^®^ 750K (SNP Affymetrix, 750K markers) performed according to the Affymetrix protocol on whole villi (cytotrophoblast and mesenchyme) showed normal results.

While waiting for results of the long-term culture, the patient was offered whole-genome sequencing-based NIPT to try to identify the marker chromosomes. A blood sample was obtained at 14.1 weeks of amenorrhea and NIPT was carried out using the VeriSeq™ NIPT Solution v2 assay (Illumina, Inc.) in the genome-wide mode as previously described (Kleinfinger et al., 2020). Following bioinformatic sequencing analysis, the NIPT results indicated a pericentromeric 29 Mb duplication of chromosome 20: dup (20) (p13q11.21) (Table 1), with a fetal fraction at 11%. As can be seen from Table 1, the “region_llr_trisomy” value was 509.27, which far exceeded the threshold value for CNVs of 15.1. In addition, a mosaic ratio of 2.06 was observed which is consistent with the presence of two extra copies and therefore suggestive of the possible presence of a tetrasomy. Based on the log-likelihood ratios, the markers appeared to be homogeneous which was concordant with the conventional cytogenetic study of the short-term culture. Subsequent targeted interphase and metaphase FISH on a short-term culture preparation of cytotrophoblast with a chromosome 20 centromeric probe [Vysis, CEP 20 (D20Z1) SpectrumOrange Probe] confirmed the segmental tetrasomy 20 in 100% of investigated cells (100/100 nuclei and 15/15 mitoses). Parental karyotypes were also performed at that time and no chromosomal anomalies were identified.

**TABLE 1:** NIPT result indicating a pericentromeric 29 Mb duplication of chromosome 20: dup (20) (p13q11.21).

Variable	Description or value
Region classification	DETECTED: dup (20) (p13q11.21)
Chromosome	Chr 20
Fetal fraction	11%
Start base	600,001
End base	29,700,000
Start cytoband	p13
End cytoband	q11.21
Region size (Mb)	29.1
Region LLR Trisomy	509.2733426
Region LLR monosomy	NA
Region t stat long reads	34.33013067
Region mosaic ratio	2.059591718
Region mosaic LLR trisomy	521.6461786
Region mosaic LLR monosomy	NA

Abbreviations: Chr, chromosome; Mb, megabase; LLR, log likelihood ratio; NA, not applicable.

**FIGURE 2:**
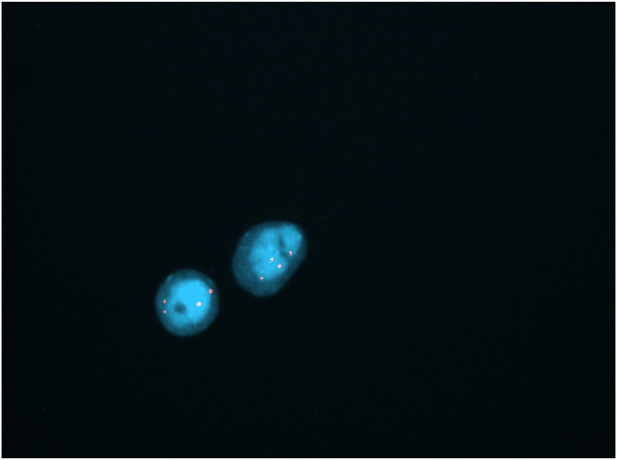
Identification of the marker chromosomes with interphasic FISH using centromeric probe of chromosome 20 (showing tetrasomy 20 in 20% of nuclei; lens 100X).

In long-term cultured villi all metaphases analyzed with conventional cytogenetics were normal (46,XX). Metaphase FISH with the 20 centromeric probe was normal in 25/25 mitoses, but interphase FISH found segmental tetrasomy 20 in 20% of the 100 examined nuclei (Figure 2). These results allowed us to conclude that this was either a case of type III confined placental mosaicism (CPM; anomaly in both the placental cytotrophoblast and the mesenchyme but not in the fetus) or type VI true fetal mosaicism (TFM; anomaly in the cytotrophoblast, mesenchyme, and the fetus).

**FIGURE 3:**
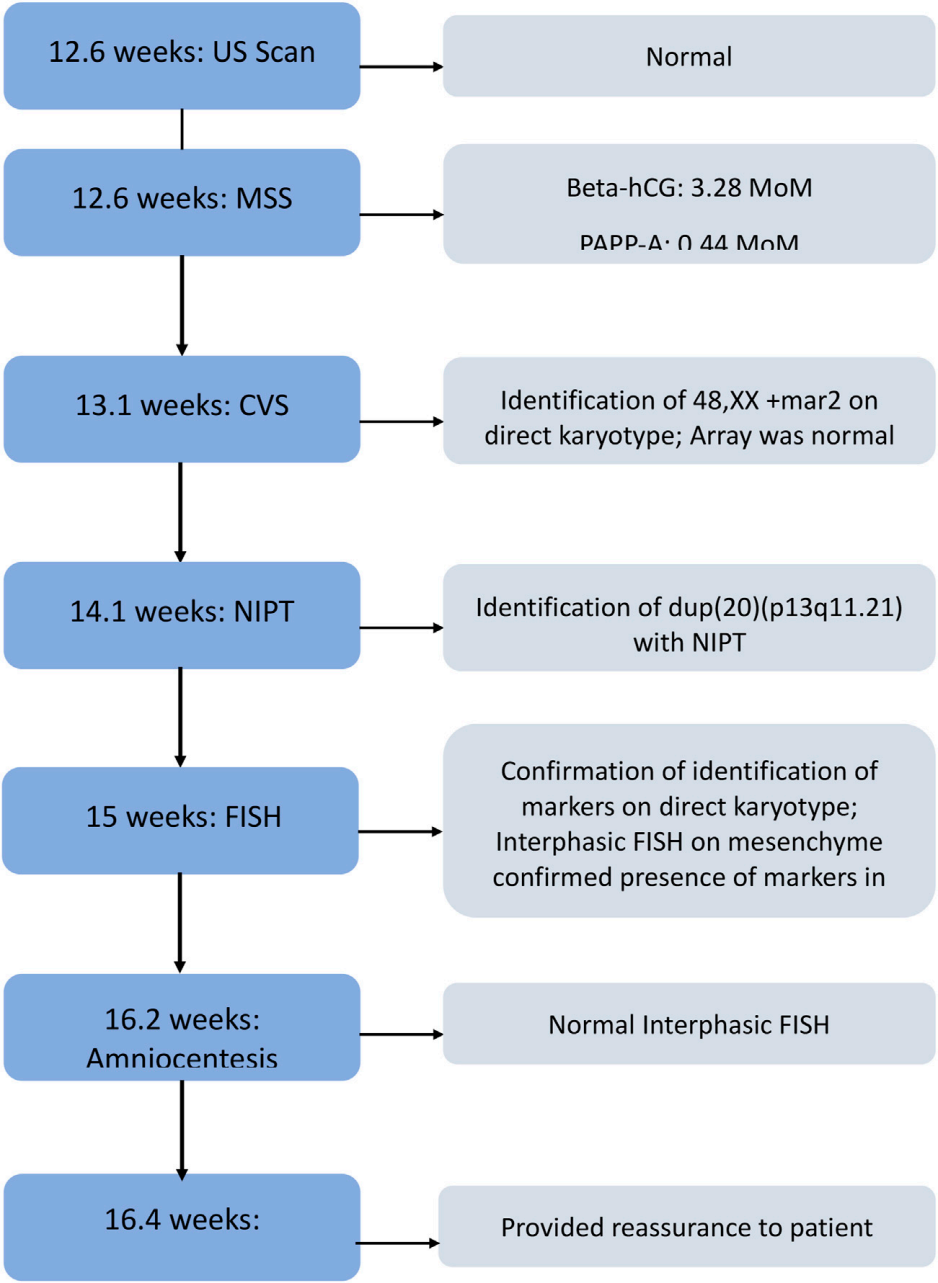
Patient’s analysis workflow by weeks of amenorrhea. US, ultrasound; MSS, maternal serum screen, CVS, chorionic villus sampling, NIPT, noninvasive prenatal testing; FISH, fluorescence *in situ* hybridization.

As both the cytotrophoblast and mesenchyme were affected, the risk was increased that the anomaly may not be confined to the placenta. To determine whether the fetus was affected, amniocentesis was performed at 16.2 weeks’ amenorrhea. Interphase FISH with the 20 centromeric probe revealed a normal result in 100/100 nuclei, allowing us to reassure the patient within 24 h of the procedure. Metaphase FISH in cultured cells was normal on 31/31 mitoses (13 clones *in situ*, 18 mitoses after trypsinization). A analysis flowchart for the patient is shown in Figure 3.

Ultrasounds carried out at 23 and 32 weeks’ amenorrhea did not show any anomalies. A normal female baby with a birth weight appropriate for gestational age was born at 40 weeks’ amenorrhea (APGAR score of 10). She presented with torticollis which spontaneously disappeared within a few days. At 1 year, she was a healthy girl, except for a G6pD deficiency (a disease with a X linked dominant transmission).

## Discussion

Small supernumerary marker chromosomes are rare; it is estimated that there are ∼3.3 million SMC carriers worldwide, of which ∼2.2 million are asymptomatic (Liehr, 2021). These marker chromosomes can originate from any of the human chromosomes. About 70% of SMCs are caused by a *de novo* event whilst 30% are inherited (Jafari-Ghahfarokhi et al., 2015). A 2007 study by Liehr and Weise found marker chromosomes to be present in 0.075% of unselected cases where prenatal diagnosis had been carried out (Liehr and Weise, 2007), and a 2014 study by Malvestiti *et al.* reported an overall *de novo* small SMC frequency of 0.072% in prenatal samples (Malvestiti et al., 2014). In addition, the clinical phenotype of SMC carriers is highly variable. It is therefore important, and also very challenging, that SMCs are characterized as soon as possible in pregnant patients to facilitate a change in pregnancy management and allow patients to make informed decisions about their pregnancy. Here, we discuss a case of a 39-year-old pregnant patient with two identical supernumerary marker chromosomes diagnosed through CVS at 13.1 weeks’ amenorrhea where additional analysis by genome-wide NIPT allowed for targeted FISH resulting in rapid, effective, and accurate characterization of the marker chromosomes and their distribution in the fetoplacental unit, ultimately allowing determination of their clinical significance.

In our case, the usual methods for identification of the markers would not have been helpful. The vast majority of SMCs are derived from acrocentric chromosomes (chromosomes 13, 14, 15, 21, and 22), with most originating from chromosome 15. Therefore, the centromeric FISH for these chromosomes takes precedence over other centromeric probes. Because the markers in our study were not from an acrocentric-derived chromosome, FISH would have been a very time-consuming approach. In addition, the SNP array failed to identify the markers. Even though array is supposed to examine both the cytotrophoblast and mesenchyme, it is not unusual for one of these tissues to be dominant. In this case, the normal result of SNP array can probably be explained by the fact that the array mainly examined the mesenchyme. Here, NIPT characterized the marker chromosomes to be pericentromeric 29 Mb duplications of chromosome 20. The risk for an abnormal phenotype in prenatally-characterized *de novo* SMC cases that are derived from a non-acrocentric autosome (such as chromosome 20) is 28% (Crolla, 1998; Liehr and Weise, 2007).

A second factor that is important in determining the clinical significance of a chromosomal anomaly is the distribution in the fetoplacental unit and the presence of mosaicism, i.e., the presence of two or more chromosomally different cell lines (Grati, 2014). As outlined above, this was a mosaic case because direct examination of CVS cytotrophoblasts showed the presence of two identical SMCs, but long-term cultures showed a normal karyotype. Identification of the markers by NIPT allowed targeted FISH analysis which found the markers in the mesenchyme, leading us to reinterpret the mosaic as either CPM type III (where the abnormal cell line is present in both the trophoblast and mesenchyme but not in the amniocytes) or TFM type VI (where the abnormal cell line is present in the trophoblast, mesenchyme, and amniocytes) (Grati, 2014).

The risk for fetal involvement is higher when mosaicism is present in both layers of the placenta compared to when it is present only in the trophoblasts (CPM type I) or only in the mesenchyme (CPM type II) (Grati, 2014). In addition, presence of the marker chromosomes in both layers of the placenta suggested that the anomaly was more likely to have originated from a meiotic error rather than a mitotic error, which puts the patient at a greater risk for pregnancy complications and UPD (Grati et al., 2021). It also increases the risk of this anomaly occurring in other pregnancies. In our case, amniocentesis was carried out at 16.2 weeks’ amenorrhea to determine the fetal karyotype. This confirmed that the marker chromosomes identified by CVS and NIPT were confined to the placenta and were not present in the fetus, allowing us to provide timely reassurance to the patient. The presence of UPD needs to be taken into consideration following prenatal identification of a marker chromosome. Although there have been a few cases reported of UPD with SMCs derived from this chromosome (Liehr et al., 2011), there is currently little to no evidence showing that UPD of chromosome 20 is associated with an abnormal phenotype and we therefore did not include UPD as a risk factor for our patient.

NIPT analyzes placental cfDNA to screen for the presence of chromosomal anomalies. This noninvasive prenatal screening test has been available for over a decade, with earlier versions of this assay typically screening for common trisomies (trisomy 21, 18, and 13) only (Nicolaides et al., 2012; Palomaki et al., 2012). Nowadays, some NIPT assays offer optional testing for a range of additional conditions including sex chromosomal aneuploidies (Mazloom et al., 2013; Samango-Sprouse et al., 2013), select microdeletion and microduplication syndromes (Helgeson et al., 2015; Martin et al., 2018), and genome-wide anomalies such as rare autosomal aneuploidies and copy number variants (Kleinfinger et al., 2020; Pertile et al., 2021; Soster et al., 2021). As shown here, genome-wide NIPT can have additional utility such as directing the choice of genetic tests/probes. Another recent case study illustrates this as well (Zhang et al., 2022). In a woman with previous failed pregnancies, results of genome-wide NIPT prompted the performance of a diagnostic test and the choice of CMA as opposed to classic karyotyping. Silver-Russell syndrome associated with a 11p15.5 duplication of maternal origin was identified; this was relevant both for decisions on additional testing in the ongoing pregnancy and also for the parents in diagnosing the cause of loss in previous pregnancies and establishing the recurrence risk. Genome-wide NIPT can play a role in identifying unbalanced chromosomal rearrangements due to parental balanced reciprocal translocations (Flowers et al., 2020).

One of the strengths of our case study was the speed at which the diagnosis was completed. In total, it took only 3 weeks from identification of the marker chromosomes on CVS, to characterization of the SMCs via NIPT and FISH, and finally analysis of the amniotic fluid to confirm that this anomaly was not present in the fetus. This prevented unnecessary extended patient anxiety. Identification and characterization of the marker chromosomes via NIPT also allowed us to avoid the high cost of multiple FISH analyses by enabling a targeted FISH approach with the appropriate probes. Finally, early identification of the type of mosaicism involved (i.e., whether this involved the cytotrophoblast, the mesenchyme, or both) was important, as this allowed us to adjust the genetic counselling that the patient received. A limitation of this study was that newborn karyotyping to confirm the absence of the markers was not performed. However, the prenatal tests on CVS and amniotic fluid allowed us to be reassured of the absence of these markers in the fetus, and the baby was healthy at 1 year of age.

In conclusion, this case illustrates that whole-genome sequencing-based cfDNA prenatal testing does not only contribute to prenatal care as a highly accurate screening test for chromosome ploidy. It can also serve as a molecular prenatal test that obviates the shortcomings of classic karyotyping and chromosomal microarray, in this case by characterizing marker chromosomes in a time- and cost-effective manner. Generating accurate and rapid results allowed for shortening the period of uncertainty for the patient and for comprehensive counseling.

## Data Availability

The original contributions presented in the study are included in the article/supplementary material, further inquiries can be directed to the corresponding author.
